# An AI healthcare ecosystem framework for Covid-19 detection and forecasting using CronaSona

**DOI:** 10.1007/s11517-024-03058-3

**Published:** 2024-03-13

**Authors:** Samah A. Z. Hassan

**Affiliations:** Information System Department, Faculty of Computers & Information, Suez University, Suez, Egypt

**Keywords:** CronaSona, COVID-19, Deep learning, COVID-19 framework, Chest X-ray, COVID-19 forecasting, COVID-19 detection, COVID-19 ecosystem

## Abstract

The primary purpose of this paper is to establish a healthcare ecosystem framework for COVID-19, CronaSona. Unlike some studies that focus solely on detection or forecasting, CronaSona aims to provide a holistic solution, for managing data and/or knowledge, incorporating detection, forecasting, expert advice, treatment recommendations, real-time tracking, and finally visualizing results. The innovation lies in creating a comprehensive healthcare ecosystem framework and an application that not only aids in COVID-19 diagnosis but also addresses broader health challenges. The main objective is to introduce a novel framework designed to simplify the development and construction of applications by standardizing essential components required for applications focused on addressing diseases. CronaSona includes two parts, which are stakeholders and shared components, and four subsystems: (1) the management information subsystem, (2) the expert subsystem, (3) the COVID-19 detection and forecasting subsystem, and (4) the mobile tracker subsystem. In the proposed framework, a CronaSona app. was built to try to put the virus under control. It is a reactive mobile application for all users, especially COVID-19 patients and doctors. It aims to provide a reliable diagnostic tool for COVID-19 using deep learning techniques, accelerating diagnosis and referral processes, and focuses on forecasting the transmission of COVID-19. It also includes a mobile tracker subsystem for monitoring potential carriers and minimizing the virus spread. It was built to compete with other applications and to help people face the COVID-19 virus. Upon receiving the proposed framework, an application was developed to validate and test the framework’s functionalities. The main aim of the developed application, CronaSona app., is to develop and test a reliable diagnostic tool using deep learning techniques to avoid increasing the spread of the disease as much as possible and to accelerate the diagnosis and referral of patients by detecting COVID-19 features from their chest X-ray images. By using CronaSona, human health is saved and stress is reduced by knowing everything about the virus. It performs with the highest accuracy, F1-score, and precision, with consecutive values of 97%, 97.6%, and 96.6%.

**Graphical Abstract:**

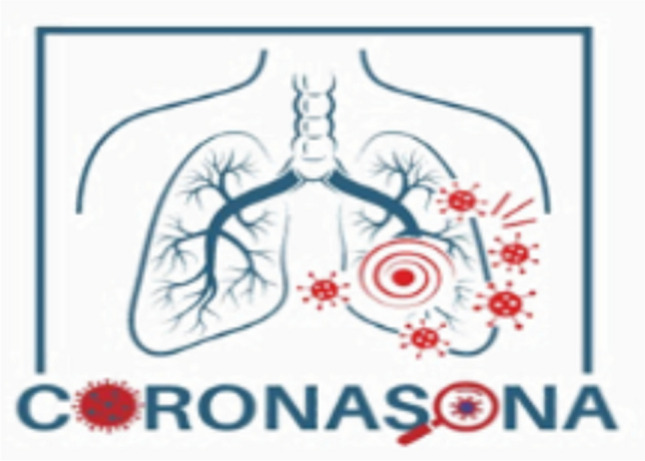

## Introduction

The world has put the COVID-19 epidemic at the forefront as it spreads fast and terrifyingly all over the world. The virus infected around 698,777,806 people, with 6,947,449 recovered and 668,915,065 deaths^1^ [1]. Addressing the epidemic requires the development of strategies to minimize infections. These strategies encompass the management and monitoring of existing cases, along with predicting the potential outcomes for individuals—be it recovery, death, or the emergence of new cases in the near future. This comprehensive approach involves collaboration across medical, engineering, and software domains.

Numerous systems, programs, and well-known websites dedicated to COVID-19 have surfaced, all with the shared objective of aiding people globally in navigating this perilous pandemic. These diverse platforms range from daily trackers, documenting the number of infections, recoveries, and fatalities worldwide, to others offering medical consultations to provide advice for individuals in quarantine.

The proposed framework, *CronaSona*, innovates by integrating diverse functionalities in one platform, providing reliable data, and fostering collaboration between stakeholders for a more robust healthcare infrastructure.

### CronaSona app

is a specialized mobile application for helping people get information about confirmed, recovered, and deceased cases. It can forecast the near future, trying to help people reduce the feeling of danger as it makes the virus statistically aware of its dimensions. It tracks patients by displaying infected people’s locations using the Google Map location tracking system and also tracks COVID-19 cases by collecting data and then generating a forecasting model to forecast near-future death, recovery, and new cases.

Through the utilization of CronaSona, the uncertainty surrounding COVID-19 no longer instills fear, as users can benefit in various ways. Firstly, users can access medical guidance within their homes, learning essential safety measures and receiving assistance. Secondly, users possess a chest X-ray checker on their devices, eliminating the need to visit hospitals and mitigating potential risks. Thirdly, users can consult CronaSona for information on available hospital beds across their country. Fourthly, users can track areas with reported cases, identifying suitable hospitals and their locations. Finally, doctors can share advice through posts, with users engaging in discussions and seeking guidance.

The framework and application are designed for scalability, future disease detection, and global use. Continuous improvement is outlined for increased stakeholder roles, advanced detection models, and generalization to detect other diseases, making *CronaSona* a potential global health application.

In this paper, Section 2 provides an overview and summarization of related work conducted by various authors. Section 3 introduces *Cronasona*, the proposed framework for COVID-19 detection and forecasting. The examination of the proposed framework is detailed in Section 4, where an intelligent mobile application, *CronaSona app*, is developed. Section 5 discusses the results, followed by conclusions in Section 6, and prospects for future work are outlined in Section 7.

## Related work

Many artificial intelligence methods and technologies are being used to detect diseases, especially in recent years, the detection of the emerging coronavirus (COVID-19).

Banja et al. [1] introduced a prototype utilizing advanced intelligent technology, aiming to provide a COVID-19 diagnosis and management solution to safeguard the well-being of individuals residing in the Kingdom of Saudi Arabia. The introduced expert system paradigms offer monitoring and control capabilities for COVID-19 patients, providing guidance to clinicians and patients using medical expertise while simultaneously alleviating the strain on healthcare providers and reducing healthcare expenses.

Asif et al. [4] proposed an optimized, lightweight shallow convolutional neural network (CNN) structure that effectively classifies patients’ X-ray images with a minimal false-negative rate. Our dataset consisted of 2541 chest X-rays. Through our model, they achieved an impressive accuracy rate of 99.68% in differentiating between healthy individuals and those infected with COVID-19. Additionally, our approach yielded excellent results in terms of precision, sensitivity, and specificity. Specifically, our proposed method demonstrated a precision of 99.66%, sensitivity of 99.66%, and specificity of 99.70%.

Oyelade et al. [5] presented CovFrameNet, a framework designed for the identification and categorization of COVID-19 presence in chest X-rays and CT images. This framework incorporates a sequential image pre-processing technique and employs a deep learning model for feature extraction, classification, and performance evaluation. The outcomes demonstrated that the proposed model achieved an accuracy rate of 0.1, recall/precision of 0.85, F-measure of 0.9, and specificity of 1.0. These results suggest that a CNN-based approach with image pre-processing capabilities holds promise for the initial screening of suspected COVID-19 cases, complementing the confirmation of COVID-19 detections based on RT-PCR methods.

Chen et al. [6] proposed a deep learning framework for the diagnosis and severity assessment of COVID-19 using chest CT scans. The framework comprises two modules: (1) the segmentation module, which extracts regions of interest and calculates the opacity percentage, and (2) the diagnosis module, responsible for identifying suspect cases and categorizing them into three groups: healthy, early stage, and progressive stage. To train and test the framework, a dataset of 150 CT exams was utilized. The obtained results include an impressive F1 score of 95.44% for COVID-19 detection, a robust F1 score of 90.87% for severity assessment, and an overall accuracy of 97.42%.

Motwani et al. [7] presented an improved framework for the automated identification and classification of COVID-19 patients using CT images. This framework utilizes a dense CNN model, transfer learning techniques, and a novel cross-entropy loss function to achieve efficient patient categorization. The proposed model has the potential to serve as a valuable clinical tool for diagnosing patients even before conducting pathogenic tests, thereby saving lives and preventing the spread of the virus. Furthermore, this model can be integrated into existing healthcare tools to accurately classify other pulmonary and chronic diseases. One significant advantage of this approach is its ability to expedite the diagnosis and treatment of COVID-19 cases. The proposed model demonstrates a prediction accuracy of 93.78% with a remarkably low false-negative rate of only 6.5%, indicating its effectiveness in diagnosing COVID-19-positive cases.

Kallel et al. [8] developed a tailored framework for monitoring and predicting the progression of COVID-19 that integrates various technologies such as machine learning (ML), cloud computing, fog computing, and the Internet of Things (IoT). This framework facilitated the collection and preprocessing of data, the implementation of classification models, and the training process using a combination of federated batch and stream ML services. Data were gathered from both medical devices (e.g., X-ray machines, lung ultrasound machines) and non-medical devices (e.g., bracelets, smartwatches) through streaming data sources. The proposed framework has the capability to incrementally learn and train from both real-time streaming data and historical stored data over time.

Pandianchery et al. [9] introduced the long short-term memory (LSTM) model for accurately predicting the number of active COVID-19 cases in each province of India. Our proposed model was trained on the data from one specific state, Maharashtra, and then tested on the data from the remaining provinces across India. The primary objective was to forecast the active cases in India from December 16, 2021, to March 5, 2022. Additionally, they experimented with various deep learning architectures, including SimpleRNN, LSTM, GRU, stacked RNN, stacked LSTM, and stacked GRU. These architectures were trained and tested on data specifically from Maharashtra. To ensure a comprehensive analysis, they conducted a detailed comparison of these architectures, considering different input window sizes and varying numbers of hidden units.

Hassan et al. [10] proposed a robust technique aimed at analyzing, predicting, and detecting COVID-19. This technique encompasses two primary tasks: infection forecasting and COVID detection. To achieve COVID detection, they employed two features: the scale invariant feature transform (SIFT) and the histogram of oriented gradients (HOG). In order to accurately predict COVID infections, they proposed a Gaussian model that facilitates reliable forecasts. For the purpose of COVID-19 detection, their technique leverages the histogram of oriented gradients and scale-invariant feature transform features. Additionally, they developed a CNN-based architecture called COVIDDetectorNet, which serves as an effective classification tool for detecting patients with COVID-19 and patients experiencing lung infections. Through our method, they achieved notable accuracy rates of 96.51% for two-class classification, 92.62% for three-class classification, and 86.53% for four-class classification.

Alruwaili et al. [11] developed a system to enhance the early diagnosis of the COVID-19 epidemic by accurately diagnosing chest X-ray images. The aim is to reduce the time required for diagnosing COVID-19 cases. Additionally, an ensemble of deep learning (DL) models is being constructed within a new framework. Furthermore, an improved DL model is being proposed, which incorporates a novel transfer learning algorithm to address the issue of overfitting, thereby making it more effective in real-time scenarios.

Ahmed and Eassa [13] have introduced a novel IoT and cloud-based blockchain model for the control of COVID-19 infection spread. This proposed model integrates IoT outdoor and indoor tracking technologies with a cloud computing-based blockchain system to effectively manage and minimize the spread of COVID-19.

Kanimozhi and Pradeep [14] proposed a framework that integrates preprocessing, feature extraction, and classification processes for the analysis of electroencephalogram (EEG) data. The preprocessing stage involves the application of principal component analysis (PCA) to eliminate artifacts from raw EEG data. Subsequently, independent component analysis (ICA) is employed for feature extraction, aiming to separate relevant features from the denoised data. The final step involves utilizing radial basis function neural network (RBFNN) for classifying the conditions based on the extracted features from the data. This comprehensive framework aims to enhance the accuracy and efficiency of EEG data analysis by systematically addressing preprocessing, feature extraction, and classification aspects.

Pradeep et al. [15] introduced a mathematical framework for modeling the rate of spread of the COVID-19 virus, utilizing an SIR-based epidemic model. Furthermore, an optimization control mechanism has been integrated into both susceptible and infected populations to regulate the spreading rate and enable early-stage diagnosis.

Table 1 between *CronaSona* and some of the previous mentioned frameworks/Models.

**Table 1:** A comparison between CronaSona and related frameworks

Ref	Framework/model	Objective/purpose	Methodology/tech	Data	Findings
[1], 2020	Expert system for COVID-19 diagnosis and management in Saudi Arabia	COVID-19 diagnosis and management, clinical guidelines	Rule-based decision support	Clinical data	Improved COVID-19 diagnosis and management
[2], 2020	Attention-based VGG-16 Model	COVID-19 detection	Attention mechanism and VGG-16	CXR images	Improved classification accuracy in COVID-19 detection
[3], 2020	Modified deep CNN for COVID-19 detection	COVID-19 detection	Xception and ResNet50V2	CXR images	Improved accuracy in detecting COVID-19 and pneumonia
[4], 2022	Deep learning framework for COVID-19 detection	Automated COVID-19 detecting	DL model	CXR images	Improved efficiency in detecting COVID-19 through imaging
[5], 2021	CovFrameNet: Enhanced deep learning framework	Enhanced COVID-19 detection	CovFrameNet	COVID-19 datasets	Enhanced accuracy in COVID-19 detection using CovFrameNet
[6], 2021	Automated COVID-19 detection and diagnosis framework	Automated COVID-19 detection and diagnosis	Dense CNN	CT scan images	Automated detection and diagnosis of COVID-19 severity
[7], 2023	Enhanced framework for COVID-19 prediction	COVID-19 prediction	Dense CNN and novel loss function	CT scan images	Enhanced framework for COVID-19 prediction using CT scan
[8], 2022	Hybrid-based framework for COVID-19 prediction	COVID-19 prediction	Hybrid federated ML models	Not specified	Enhanced COVID-19 prediction via hybrid framework
[9], 2022	Explainable AI framework for COVID-19 prediction	COVID-19 prediction in India	Explainable AI framework with interpretability	Indian COVID-19 data	Explainable AI for improved COVID-19 prediction in India
[10], 2022	A robust framework for epidemic analysis, prediction and detection	COVID-19 detection epidemic analysis and prediction, and detection	Robust epidemic analysis framework	COVID-19 data	Improved epidemic analysis, prediction, and detection of COVID-19
[11], 2021	COVID-19 diagnosis model	COVID-19 diagnosis	Enhanced inception-ResNetV2	CXR images	Improved COVID-19 diagnosis using enhanced Inception-ResNetV2
[13], 2022	COVID-19 infection spread control model	COVID-19 infection spread control	IoT, cloud, blockchain	Not specified	Improved infection spread control
[15], 2021	Optimized SIR modeling for analyzing Covid-19 spreading rate	COVID-19 spreading rate analysis	Optimized SIR modeling framework	COVID-19 data	Analysis of spreading rate
CronaSona	AI ecosystem framework for Covid-19 detection and forecasting	COVID-19 diagnostic tool (detection, forecasting, and tracking)	CNNs, flutter, SQLite, Google Maps, TensorFlow, Keras, and forecasting model (FBPROPHET)	CXR images, COVID-19 data, Google Maps, JSON file, KB and DB	Improved and accelerated COVID-19 detection, diagnosis, forecasting, tracking, and awareness

The previous works encountered several challenges and limitations in one or more of the following:

- The absence of a collaborative approach among academic, research, healthcare, and governmental institutions may have limited the overall impact of previous works.
- Certain frameworks and models demonstrated shortcomings in achieving high diagnostic accuracy and performance when it comes to detecting COVID-19 cases from chest X-ray images, particularly in the early stages of infection.
- Some frameworks may not have employed robust forecasting models, affecting their ability to predict COVID-19 spread accurately.
- Certain frameworks may have faced challenges in adopting and integrating emerging technologies, such as deep learning models and forecasting tools.
- The integration of different subsystems and components, as well as interoperability with existing healthcare systems, could be a challenge in some frameworks.
- Previous works missed the communication and interaction mechanism among administrators, healthcare providers, and users could hinder the overall functionality of the system.
- The level of user engagement and interaction in certain frameworks may have been insufficient.
- Many existing frameworks and applications lacked scalability, making it difficult to handle a large volume of users and data.
- Some frameworks were designed for specific regions or populations, limiting their generalizability to diverse demographic groups and global applicability.
- Some frameworks may not have covered the entire healthcare ecosystem, leaving gaps in areas such as mobile tracking, expert advice, and comprehensive management information.

## Materials and methods

**Fig. 1:**
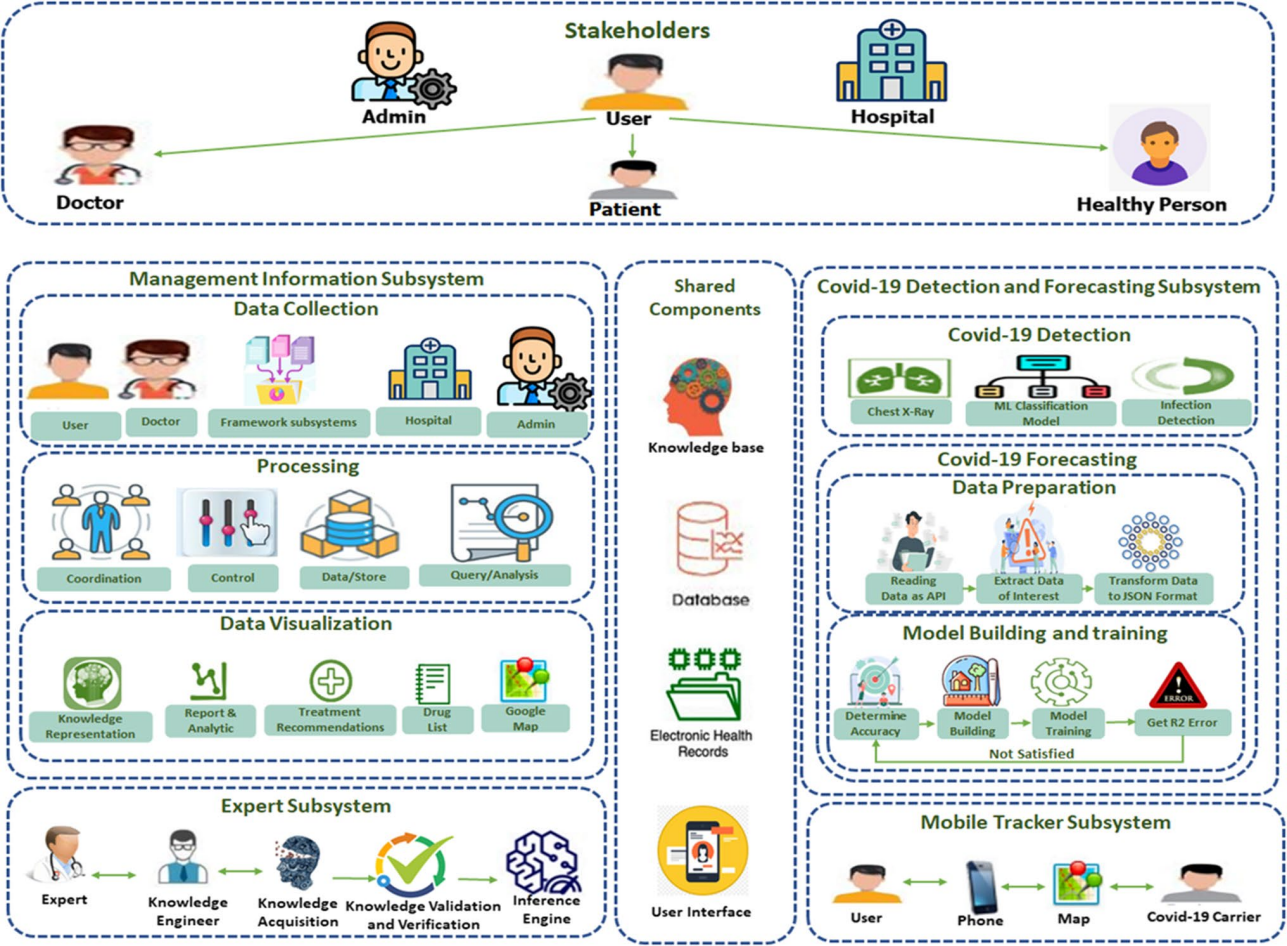
The proposed COVID-19 ecosystem framework

This section covers the conventional procedure for the proposed framework, *CronaSona*. The strategy involves initial data preprocessing, followed by the utilization of a classification model derived from the convolutional neural network (CNN) model. The dataset utilized in this study is described. The proposed approach is initially depicted in Fig. 1. The framework parts are (1) stakeholder’s part and (2) shared components part, while its subsystems are (1) management information, (2) COVID-19 detection and prediction, (3) mobile tracker, and (4) expert subsystem.

### CronaSona parts

A. Stakeholder part

The stakeholders’ part includes all types of system users. It encompasses a diverse array of system users, each playing distinct roles within the system. These stakeholders include administrators, users (comprising doctors, patients, and healthy individuals), and subscribed hospitals. These stakeholders collectively form a network that supports efficient healthcare management, diagnosis, and communication within the CronaSona ecosystem. The system facilitates seamless collaboration among diverse user roles, contributing to effective pandemic response and healthcare delivery. Table 2 explores the roles and responsibilities of each stakeholder category.

**Table 2:** Stakeholder roles and responsibilities

Stakeholder		Roles	Responsibilities
Administrators		Oversee and manage the overall CronaSona functionalities	- Manipulate CronaSona data and approve accepted data - Assist in the manipulation of hospital-related data, especially concerning available rooms - Facilitate communication between various stakeholders - Oversee and ensure the proper functioning of the entire system
Users	Doctors	Provide medical expertise and guidance	- Check and access detailed information about patients - Communicate with patients through posts and comments - Provide medical advice and recommendations based on uploaded tests and X-ray images
	Patients	Contribute their medical data for diagnosis and treatment	- Upload X-ray images and other relevant medical tests - Access drug lists and treatment plans - Communicate with doctors through posts and comments - Track nearby patients for awareness - Check available hospitals
	Healthy User	Utilize the system for preventive measures and awareness	- Check available hospitals and their details - Communicate with doctors for general health queries - Track nearby patients for proactive measures - Make informed decisions about places to visit
	Hospitals	Contribute to the collective healthcare network	- Provide and update information about available rooms - Subscribe to the system to enhance healthcare services - Collaborate with administrators to manage hospital-related data - Collaborate with other stakeholders for effective patient care

B. Shared component part

Shared components collectively form the backbone of CronaSona, ensuring that data is appropriately managed, shared, and utilized across different parts of the healthcare ecosystem. It plays a crucial role in facilitating seamless interactions and data management among various subsystems. The integration of these components enhances the overall user experience and contributes to the successful implementation of the proposed framework. It includes four components, which are (1) a* knowledgebase* that was collected mainly from two resources: the expert subsystem and the COVID-19 detection and forecasting subsystem. It contains knowledge about doctors’ instructions, classified chest X-ray images, and rules discovered from the previous patients’ electronic health records. (2) Database, which serves as a repository for stakeholder-related information, encompassing data such as user profiles, personal details, patient records, medication lists, hospitalization details, contact lists of patients, doctors’ interactions with patients, treatment plans, and management protocols. (3) Electronic health record, which includes patients’ reports and/or their chest X-ray. Stakeholders will communicate and/or interact with the framework subsystems through (4) *the user interface*. Table 3 summarizes the shared components part.

**Table 3:** The shared component parts

Component	Description	Contents
Knowledgebase	Serves as a repository for essential information, primarily derived from the Expert Subsystem and the COVID-19 Detection and Forecasting Subsystem	- JSON files containing doctors’ instructions, - information on confirmed, recovered, and deceased cases, - Other relevant medical knowledge
Database	A storage facility for diverse data related to stakeholders, their interactions, and crucial information necessary for the functioning of CronaSona	Stored data includes: - user profiles, - communication posts, - comments, - drug lists, - details about patients and healthcare providers
Electronic Health Record (EHR)	A centralized repository for storing detailed information related to patients' health, including uploaded data, tests, and chest X-ray images	- Patient records - diagnostic tests - medical images
User interface	Encompasses the various forms and pages that users interact with when using the CronaSona application. It facilitates user engagement and system navigation	Interactive features as: - ConaSona forms; registration/login, - information retrieval, - obtaining drug lists

### CronaSona subsystems

A. Management information subsystem

The management information subsystem within the CronaSona framework plays a vital role in collecting, processing, and presenting data gathered from various sources. It collects data from a range of sources, processes it, and presents it in a readable format for decision-making. It is responsible for managing information related to stakeholders. The main role is to manage collected data and disseminate information to other subsystems. In the *data collection phase*, data is collected from stakeholders and/or other subsystems. The collected data includes users’ personal data, laboratory tests, and X-ray images provided by patients, supportive care, and treatment recommendations provided by doctors, hospitals’ details and drug lists entered by admins, image classification and forecasting results provided by the COVID-19 detection and forecasting subsystem, and nearby carriers provided by the mobile tracker subsystem. According to the nature of the collected data, it is stored in a knowledge base, database, or electronic health record on the shared component’s part. The *processing phase* includes rules and procedures associated with coordination between users, control, storing data, querying information, and/or analyzing results. Users have the ability to make inquiries or retrieve information from both the database and the knowledgebase. The *data visualization phase* includes knowledge representation, reports and analysis of results, treatment recommendations from doctors, drug lists for patients, or maps to represent nearby carriers.

B. Expert subsystem

The expert subsystem in CronaSona incorporates a life cycle approach to knowledge base development, including knowledge acquisition, validation, and verification. This systematic approach enhances the reliability of expert advice provided within the application. The development of the knowledge base goes through a life cycle comprising three phases: (1) knowledge acquisition, (2) knowledge verification and validation, and (3) the implementation of the inference engine. The knowledge can be collected either from expertise or from the detection and forecasting subsystem. After the information is collected, it needs to be revised by a knowledge engineer. The expert subsystem includes the following components:

- An expert is a person who has special skills and/or knowledge in the medical field, such as doctors.
- Knowledge engineer is an AI language and knowledge representation expert responsible for executing the life cycle of knowledge base development. Their role involves exploring a specific problem domain, identifying crucial concepts, and creating accurate and efficient representations of the objects and relationships within that domain.
- Knowledge acquisition refers to the systematic procedure of extracting, structuring, and organizing knowledge, typically obtained from human experts. This process involves adding new knowledge to a knowledge base as well as refining or enhancing previously acquired knowledge. Over time, this continual acquisition and refinement of knowledge contributes to improved accuracy and more reliable outcomes.
- Knowledge validation and verification: Knowledge validation and verification focus on quality assurance to avoid inconsistencies or mistakes by revising knowledge to assure that it is validated and verified. The process can involve leveraging an odd number of experts to resolve any disagreements through majority voting. Following a thorough review of the knowledge, all comments are carefully addressed to ensure correctness. Once verified, the knowledge is then incorporated into the knowledge base.
- Inference engine: The inference is made on the knowledge base by applying logical rules to figure out new facts and/or information.

C. COVID-19 detection and forecasting subsystem

This subsystem is responsible for detecting COVID-19 carriers from their chest X-rays and forecasting the near future—the next 7 days—death, recovery, and new cases. It includes the following two phases:

- The first phase, COVID-19 detection

According to this phase, a ML classification model is used to classify users’ chest X-rays. The classification result will be either normal or infected, depending on the infection percentage. Firstly, a user will input a chest X-ray image to the subsystem. Secondly, DL classification models will be used to process and classify this image in order to detect the degree of virus infection. Lastly, according to the classification model, the result will be displayed. It helps the patient get the closest possible result by displaying the percentage of infection with COVID-19.

**Algorithm 1:**
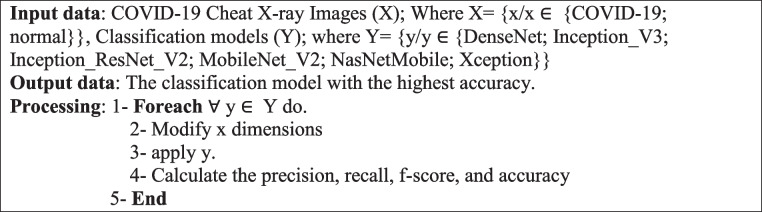
Detection Module

**Fig. 2:**
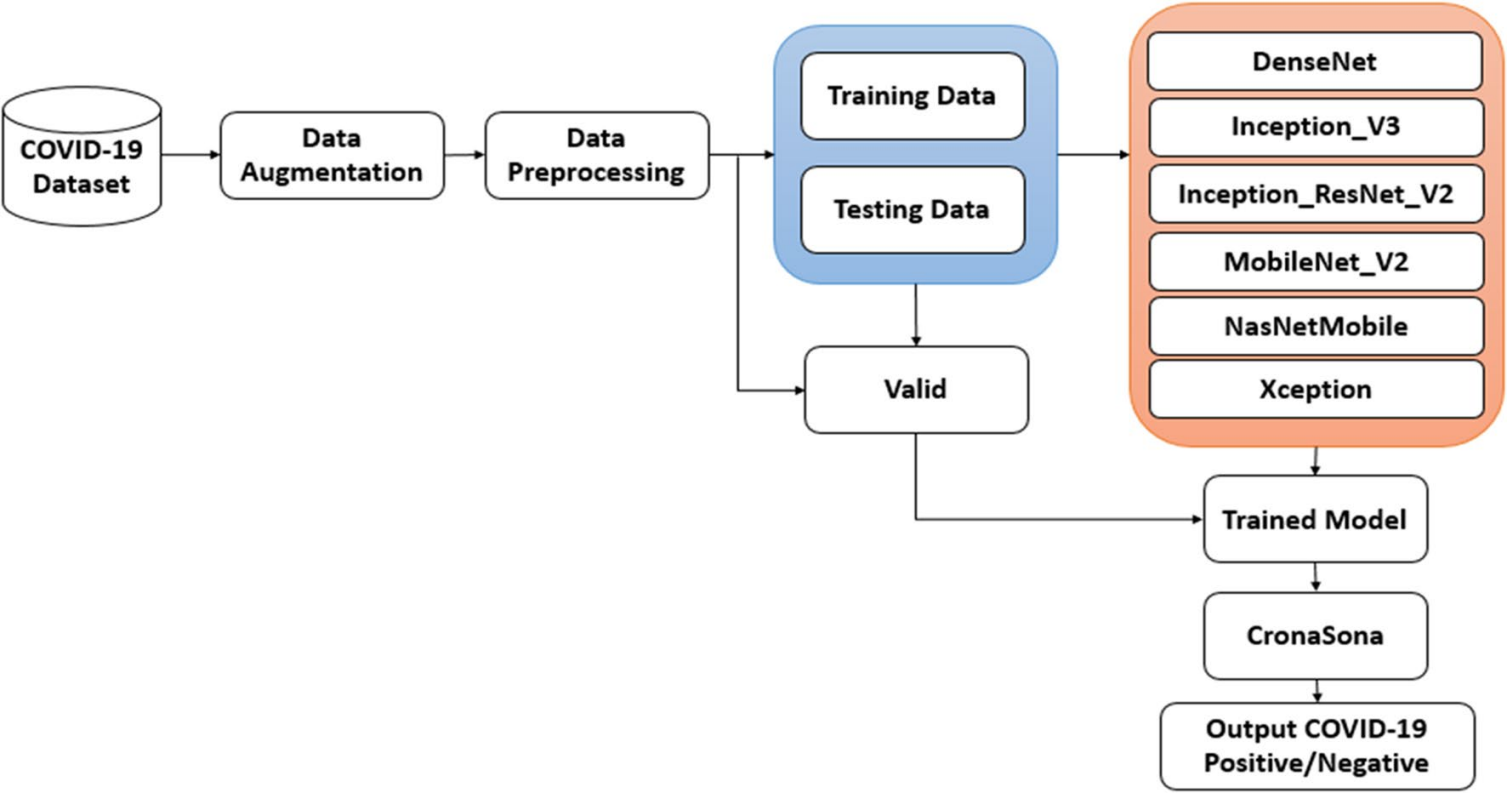
COVID-19 prediction model (CronaSona)

The subsystem of the proposed model is depicted in Fig. 2 encompasses a sequence of six steps [16]. These steps entail (1) collecting the dataset, (2) implementing data augmentation, (3) conducting data preprocessing, (4) establishing training and testing sets, (5) training and optimization, and (6) evaluations for assessing the overall performance.

1. Dataset collection and description

The sample of chest X-ray images covered two main classes: COVID-19 for positive cases and negative for healthy cases. The chest X-ray images are obtained from the COVID-19 Radiography Dataset.^2^ It contains a total of 5863 X-ray images (JPEG), which are distributed into three folders: 624 images for testing, 5216 images for training, and 16 images for validation, each with two categories: pneumonia for COVID-19 images and normal for healthy images, as shown in Table 4.

**Table 4:** The distribution of testing, training, and validation sets used in the dataset

	No. of images	Test	Train	Val
Normal	1583	234	1341	8
Pneumonia	4273	390	3875	8

As seen from Table 4, the distribution of images in the dataset is not suitable because it lacks balance among the classes, particularly in the test, training, and validation sets. For instance, in a medical imaging dataset with two classes (normal and pneumonia), having a highly imbalanced distribution can lead to biased model training and evaluation. In this case, there are substantially more pneumonia images than normal images in the training set, which may cause the model to be biased towards predicting pneumonia, affecting its generalization to new, unseen data.

To address this issue, data augmentation techniques are suggested. Data augmentation involves creating additional training samples by applying various transformations to the existing images. This helps in diversifying the dataset and improving the model’s ability to generalize to different scenarios. The proposed approach mentions the use of multiple data augmentation techniques, such as flipping and rotation, to accurately capture and comprehend the various transformations that occur during the training phase of deep learning models.

2. Data augmentation

The application of data augmentation is a commonly employed method to increase the variety and volume of annotated training datasets by generating additional training samples through applying various transformations. The aforementioned objective is accomplished by the implementation of input transformations that preserve the corresponding output labels. The objective of this approach is to enhance the overall performance and outcomes of machine learning models by increasing the quantity of accessible data. Furthermore, varieties of photo augmentation techniques are utilized to enhance images within the realm of deep learning. The proposed approach integrates multiple data augmentation techniques, such as flipping and rotation, in order to accurately capture and comprehend the various transformations that occur during the training phase of the DL Models. Table 5 shows a comparison between the used data augmentation techniques, Flipping and Rotation.

**Table 5:** A comparison between data augmentation techniques (flipping and rotation)

Aspect	Flipping	Rotation
Description	Simulate changes in the viewpoint from left to right (horizontally), from top to bottom (vertically), or combines horizontal and vertical flipping (diagonal flip)	Rotating the image at various angles, usually in increments of 90° or arbitrary angles. Allows the model to learn robust features from images with different viewpoints
Implementation complexity	Simple to implement	Requires more complex calculations
Effect on image size	No change in image dimensions	May result in a larger bounding box
Purpose	It is commonly applied in tasks like object detection, where the object’s appearance is invariant to horizontal or vertical flips	It is helpful in tasks like image classification, where objects may appear at different angles
Impact on model generalization	Can help improve model generalization by exposing it to mirrored versions of objects	Offers a broader view, aiding the model in recognizing objects from different angles
Advantages	- Simple and computationally efficient - Helps the model learn variations in orientation	- Provides a broader range of transformations - Helps the model generalize to different perspectives
Disadvantage	- May not be suitable for all image types, especially those with distinct directional features - May introduce unrealistic variations	- May increase computational complexity - May introduce distortion, and large rotations could result in loss of information

3. Data preprocessing

The COVID-19 detection model being considered includes a preprocessing stage that is largely focused on enhancing the quality of the input image. The implementation of the preprocessing stage aims to address undesirable distortions and improve specific qualities present in the image. The procedure of image enhancement entails mitigating the adverse impacts of blurriness or noise present in the input image. The preprocessing stage involves the utilization of two approaches, specifically image scaling using the Nearest-Neighbor Interpolation technique, which is fast and computationally less intensive, and median filtering using the standard median dilter technique, which is effective for salt-and-pepper noise reduction. The process of image scaling is predominantly efficient in the resizing of input chest X-ray pictures. It is crucial in this operation to ensure that the quality of the input image remains unaffected by any form of degradation. The utilization of median filtering is employed to generate an image that is devoid of noise. The procedure entails the utilization of a non-linear filter, wherein the median value is employed to maintain the clarity of the image, particularly at its edges. The median value restoration has been accomplished by use gray level ranking instead of the noise value.

There are several libraries and modules that can be used to read and preprocess JPEG images, especially when working with Python and TensorFlow as a deep learning framework. For example, “tensorflow.image.resize” is used to resize images and “tensorflow.image.convert_image_dtype” is used to convert the image data type to a suitable format to be used by various DL models. Table 6 shows a comparison between image scaling and median filtering.

**Table 6:** A comparison between data preprocessing techniques (scaling and median filtering)

Aspect	Image scaling	Median filtering
Description	Adjusts the size of an image, scaling up (enlarging) and scaling down (reducing), while maintaining its aspect ratio	A noise reduction technique that replaces each pixel’s value with the median value of its neighborhood
Objective	Concerned with adjusting the size of an image	Focused on noise reduction
Usage	Often used as a preprocessing step, in preparing datasets, to standardize input sizes	Applied before feature extraction to enhance the quality of images by reducing noise
Effect on image	Alters the physical size of the image	Smoothes the image by reducing noise
Implementation complexity	Fast and computationally inexpensive	Depends on the size of the neighborhood; larger neighborhoods require more processing
Advantages	- Commonly applied in convolutional neural networks (CNNs) for image classification tasks - Useful for preparing datasets for deep learning models, ensuring uniform input sizes	- Suitable for preprocessing images with noise, especially where noise reduction is essential such as medical imaging or low-light conditions - Improve the clarity of edges in images and is effective against salt-and-pepper noise
Disadvantages	- Scaling up may result in loss of detail - Scaling down may lose information	- May blur fine details - Less effective for certain types of noise

4. Establishing training and testing sets.

In the preliminary stage, data augmentation was utilized to tackle the challenge of imbalanced sample distribution in the raw X-ray images. After the augmentation process, the improved dataset is then partitioned into three separate subsets, namely the train, test, and validation sets.

5. Training and optimization.

During the training and optimization phase, the CronaSona network model has been employed and requires fine-tuning to accurately identify the COVID-19 classes as either positive or negative. Hence, the model is optimized to achieve the most optimal decision-making model. The utilization of a validation error has been employed to enhance the optimization process. Following this, the optimal model has been acquired and subsequently employed in the decision-making procedure using the test dataset. The optimal network model achieved successfully classifies the different classes of COVID-19 and accurately evaluates the performance of the classification.

6. Evaluations.

The assessment of the predictive capabilities and effectiveness of the provided prediction models entails the application of five metrics. The evaluation of each model’s performance involves the employment of many measures, such as accuracy, F-measure, recall, and precision [12]. The determination of accuracy is achieved through the utilization of the following equation.

1 $${\text{Accuracy}}=\frac{{\text{TPos}}+{\text{TNeg}}}{{\text{TPos}}+{\text{FPos}}+{\text{FNeg}}+{\text{TNeg}}}$$

where TPos is true positive, Tneg is true negative, Fpos is false positive, and Fneg is false negative.

Precision is calculated using the following equation:

2 $${\text{Precision}}=\frac{{\text{TPos}}}{{\text{TPos}}+{\text{FPos}}}$$

Recall is computed using the following:

3 $${\text{Recall}}=\frac{{\text{TPos}}}{{\text{TPos}}+{\text{FNeg}}}$$

F-measure is calculated using:

4 $${\text{F}}-{\text{measure}}=\frac{2\times {\text{Recall}}\times {\text{Precision}}}{{\text{Recall}}+{\text{Precision}}}$$

- The second phase, COVID-19 Forecasting:

This phase is divided into two stages. According to the first stage, *data preparation*, the subsystem will (1) read data from its source, the World Health Organization, as a JSON file; (2) extract data of interest; and (3) transform data to JSON format. In the second stage, ***model building and training***, (1) the result accuracy is needed to be determined. (2) The forecasting model will be built. (3) The model will be trained. (4) The R2 error will determine whether to repeat this stage or not.

**Algorithm 2:**
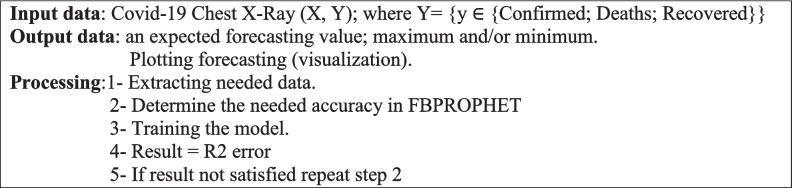
Forecasting Module

D. Mobile tracker subsystem

An integrated mobile tracker subsystem is implemented to monitor individuals who may be carriers of COVID-19 by tracking their locations on a map. This system assists healthy individuals by providing the ability to check if COVID-19 carriers have been present at specific locations, thus enabling proactive measures. By displaying the locations of these carriers, the system aids in isolating them from healthy individuals and minimizing the potential spread of the virus.

The integrated subsystem utilizes Google Maps and a phone number list of confirmed COVID-19 cases to locate individuals, allowing for the identification of places they have recently visited. This information is then used to notify those who may have been exposed to the virus at these specific locations. By displaying a Google map, it becomes possible to highlight areas where suspected cases have occurred, thereby enabling healthy individuals to make informed decisions about the places they choose to visit.

Table 7 compares and highlights the key aspects of the management information subsystem, expert subsystem, COVID-19 detection and forecasting subsystem, and mobile tracker subsystem within the CronaSona framework:

**Table 7:** A comparison between CronaSona framework subsystems

Subsystem	Role	Data source	Data processing	User interaction	Integration with other subsystems
Management information subsystem	- Collects, processes, and presents data - Manages collected data and disseminates information to other subsystems	Stakeholders and other subsystems	Coordination between users, control, storing data, querying information, analyzing results	Inquiries, information retrieval, data visualization	Collaborates with COVID-19 detection and forecasting subsystem, mobile tracker subsystem, and shared components
Expert subsystem	- Develops knowledge base through knowledge acquisition, verification, validation, and inference	Expertise or detection and forecasting subsystem	Exploration of problem domain, identifying concepts, creating representations Extraction, structuring, organizing knowledge; Validation and verification	Knowledge acquisition, validation, verification, inference	Collaborates with management information subsystem
COVID-19 detection and forecasting subsystem	- Detection: ML classification of chest X-rays - Forecasting: Model building and training	Users’ chest X-rays, World Health Organization (WHO) data	ML classification, forecasting model building, training, R2 error determination	Input of chest x-ray images; display of infection percentage	Collaborates with management information subsystem, expert subsystem
Mobile tracker subsystem	- Monitoring carriers’ locations on a map - Notification for potential exposure	Google Maps, phone number list of confirmed COVID-19 cases	Location tracking, identification of visited places	Checking exposure, notifies individuals, making informed decisions	Collaborates with management information subsystem

## Results and discussion

**Fig. 3:**
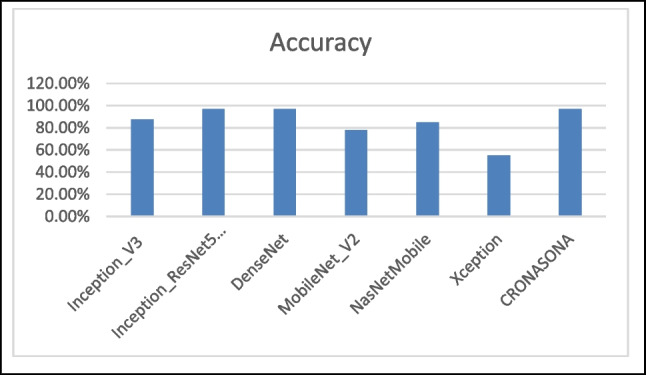
A comparison between the used DL models vs. CronaSona in terms of accuracy

The subsequent section showcases the simulation outcomes of the proposed ideal model for forecasting the transmission of COVID-19. The implementation of the machine learning models was performed using the Jupyter Notebook version 6.4.6. The use of Jupyter Notebook enables the streamlining of Python code writing and execution, hence augmenting productivity and convenience. The assessment of the predictive accuracy is carried out by comparing the results obtained from the proposed model with those generated by other state-of-the-art deep learning algorithms. In addition, the assessment of the effectiveness of the proposed methodology frequently entails the application of characteristic curves and precision measures, commonly utilized to measure the performance of machine learning models. These metrics provide quantitative measures of the model’s efficacy in accurately classifying instances, identifying true positives, true negatives, false positives, and false negatives, and evaluating the overall performance of the model. The accuracy metric measures the proportion of correctly classified cases relative to the total number of instances. Sensitivity, also known as recall or true positive rate, is the proportion of true positives accurately identified by the model. The accuracy findings obtained from the experimental investigation are presented in Table 5. Among all of the experimental models shown in Table 8, CronaSona model shows superior accuracy compared to other models. Specifically, its accuracy, F1-Score, Recall, and precision are 97%, 97.6%, 89% and 96.6%, respectively as shown in Fig. 3.

**Table 8:** Model classification results for two-category (COVID-19 vs. normal) classification task for the dataset

Model	TP	FP	TN	FN	Precision	Recall	F-score	Accuracy
Inception_V3	165	9	381	69	84.67%	97.69%	90.71%	87.5%
Inception_ResNet50_V2	199	5	385	35	92%	99%	95%	96.85%
DenseNet	170	6	384	64	85.7%	98.5%	91.6%	96.7%
MobileNet_V2	40	71	319	194	62%	82%	71%	78%
NasNetMobile	50	82	308	184	60%	67%	63%	85%
Xception	82	127	263	152	63%	67%	65%	55%
CronaSona	200	5	395	30	96.6%	89%	97.9%	97%

**Fig. 4:**
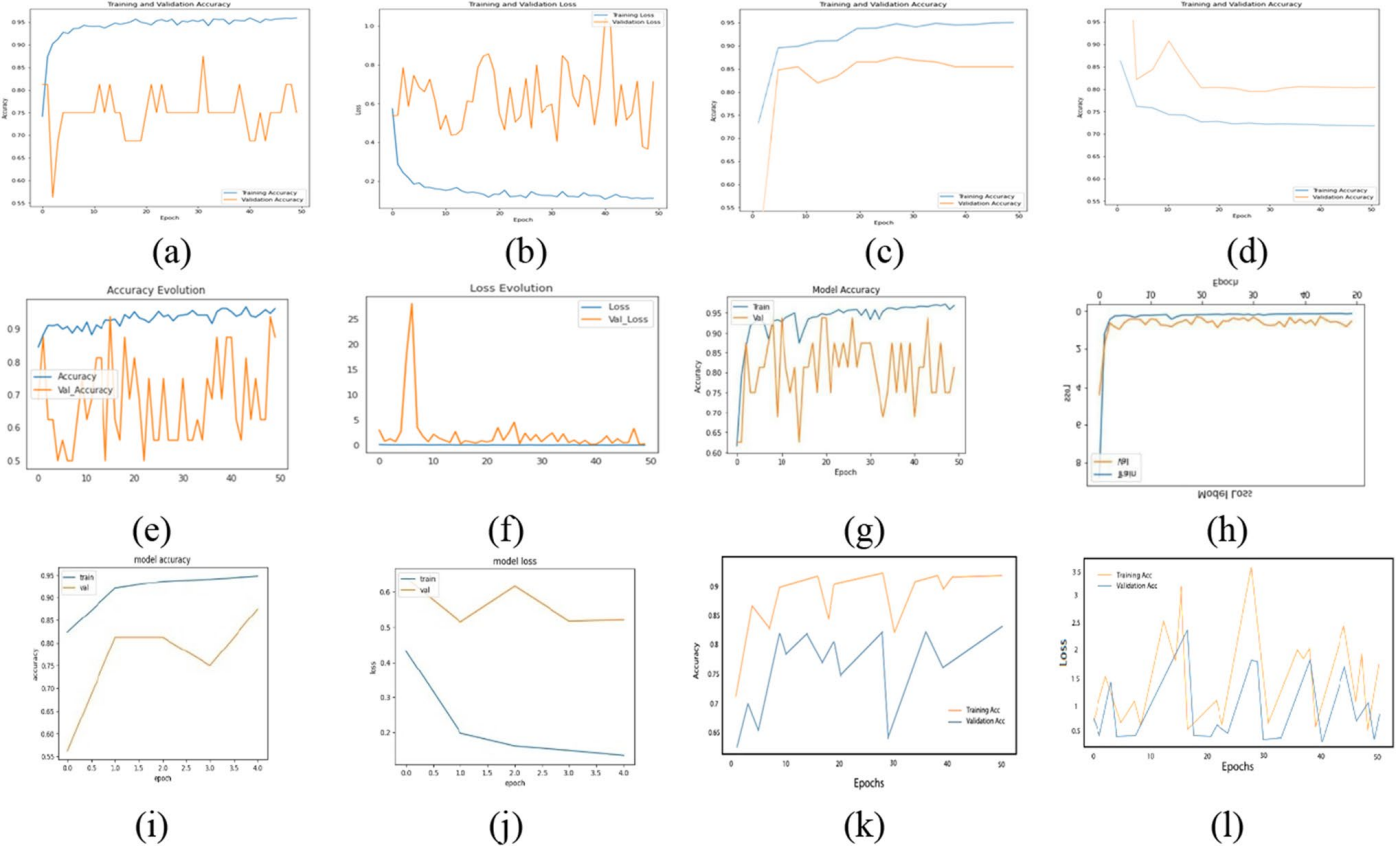
The applied five DL classification model accuracy and loss for each epoch. **a** Inception_V3 accuracy, **b** Inception_V3 loss, **c** Inception_ResNet50_V2 accuracy, **d** Inception_ResNet50_V2 loss, **e** DenseNet accuracy, **f** DenseNet loss, **g** MobileNet_V2 accuracy, **h** MobileNet_V2 loss, **i** NasNetMobile accuracy, **j** NasNetMobile loss, **k** Xception accuracy, and **l** Xception loss

Figure 4 shows the selected 6 DL classification models accuracy vs. epoch and loos vs. epoch.

**Fig. 5:**
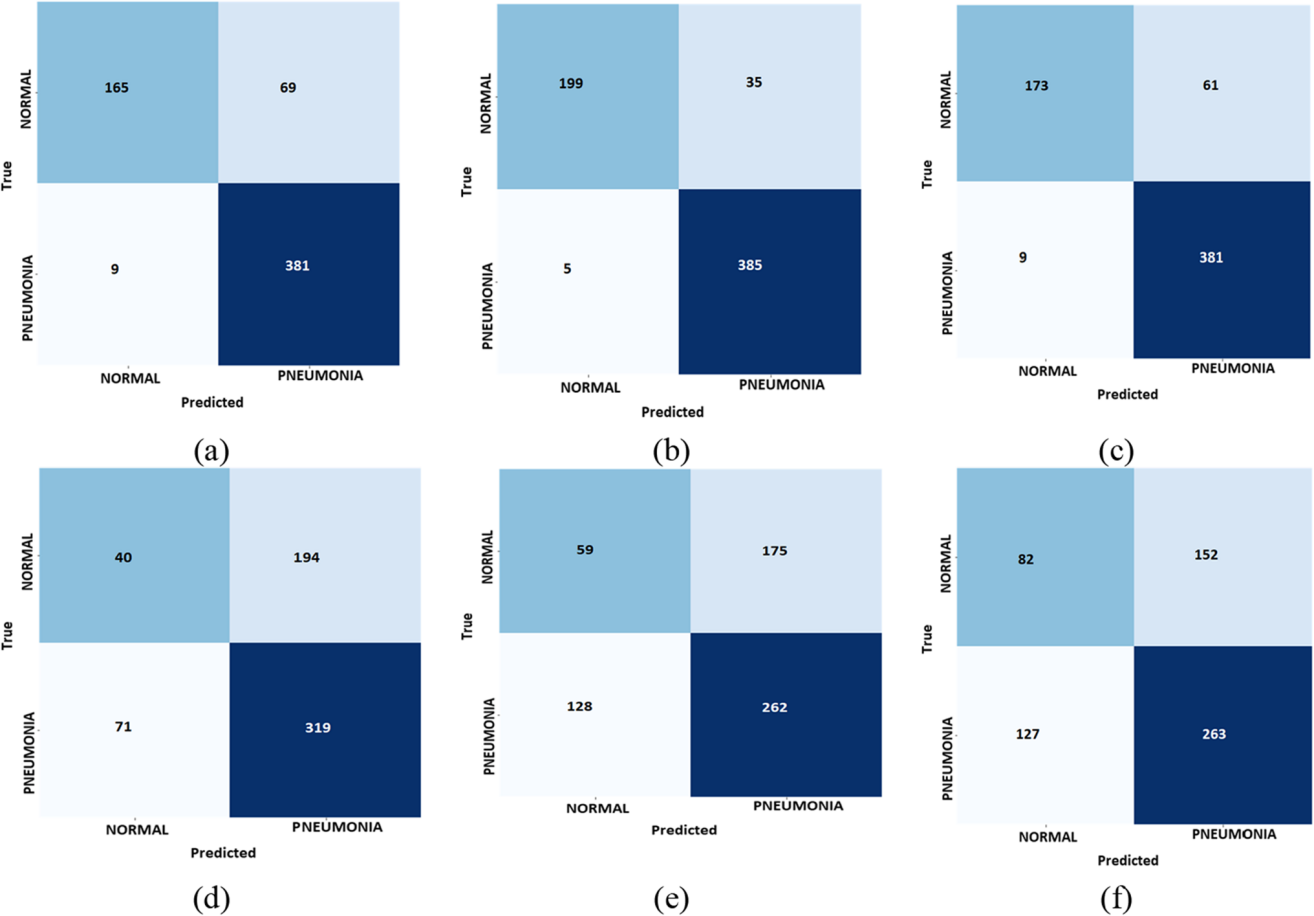
The confusion matrix of the six models for COVID-19, normal, respectively. **a** Inception_V3, **b** ResNet50_V2, **c** DenseNet, **d** MobileNet_V2, **e** NasNetMobile, and **f** Xception

The following Fig. 5 shows the confusion matrix of the 6 DL classification models VS. CronaSona.

## Applying cronaSona

CronaSona was built to test the proposed framework functionalities. The aim of this application is to develop and test a reliable diagnostic tool using deep learning techniques to detect COVID-19 features from chest X-ray images. CronaSona would accelerate the diagnosis and referral of patients. This particular application also has the potential to be scaled up and used for more generalized, high-impact applications in biomedical imaging. Table 9 shows the main CronaSona functionalities.

**Table 9:** CronaSona functionalities

Framework part		Usage/functionalities
Stakeholders	Admin	- Manipulate CronaSona data; especially approve accepted data
	Hospital	- Manipulate hospital data; especially available rooms
	Doctor	- Check patients’ details - Communicate patients post/comment
	Patient	- Upload X-ray/tests - Get drug list - Check available hospitals - Communicate doctors’ post/comment - Track nearby patients
Shared components	*Knowledgebase*	- JSON files which contains doctors’ instructions - JSON file for the confirmed, recovered, and dead cases
	*Database*	- Includes saved data about stakeholders, and their communication posts and/or comments - Data about drug list
	*Electronic health record*	- Includes patients’ uploaded data, tests, and/or x-ray images
	*User Interface*	- Forms and pages used to interact with CronaSona app
Management information subsystem		- Includes forms for registration, login, information retrieval, getting drug list
Expert subsystem		- Doctors’ advices and comments on patients’ uploaded tests/X-ray images
COVID-19 detection and forecasting subsystem		- X-ray image detection - Forecasting
Mobile tracker subsystem		- Provide a map to track nearby infected patients

Flutter is used as a cross-platform to develop CronaSona. In Flutter, the Scaffold class offers a range of widgets and APIs such as Drawer, SnackBar, BottomNavigationBar, FloatingActionButton, and AppBar. It serves as a foundational component that facilitates the implementation of the application’s basic material design layout. SQLite is used as a tool to build the CronaSona database. In order to build the mobile tracker subsystem, Google Maps is used through APIs. Tensorflow is used to run the training process for the classification models. Keras is chosen as an open-source, high-level deep neural network library to run on TensorFlow. Fbprophet is chosen as an open-source prophet for the forecasting process. It is a forecasting tool that Facebook developed and is available in both Python and R. The forecasting process is done based on data given from a JSON file, such as the following:
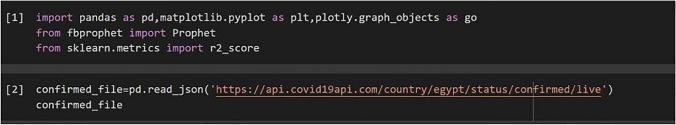


The following code is used to training the dataset with performance accuracy 0.97.
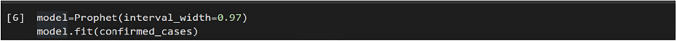


The following code is used for forecasting the next week day by day.
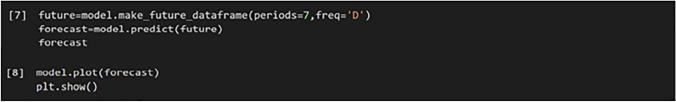


**Fig. 6:**
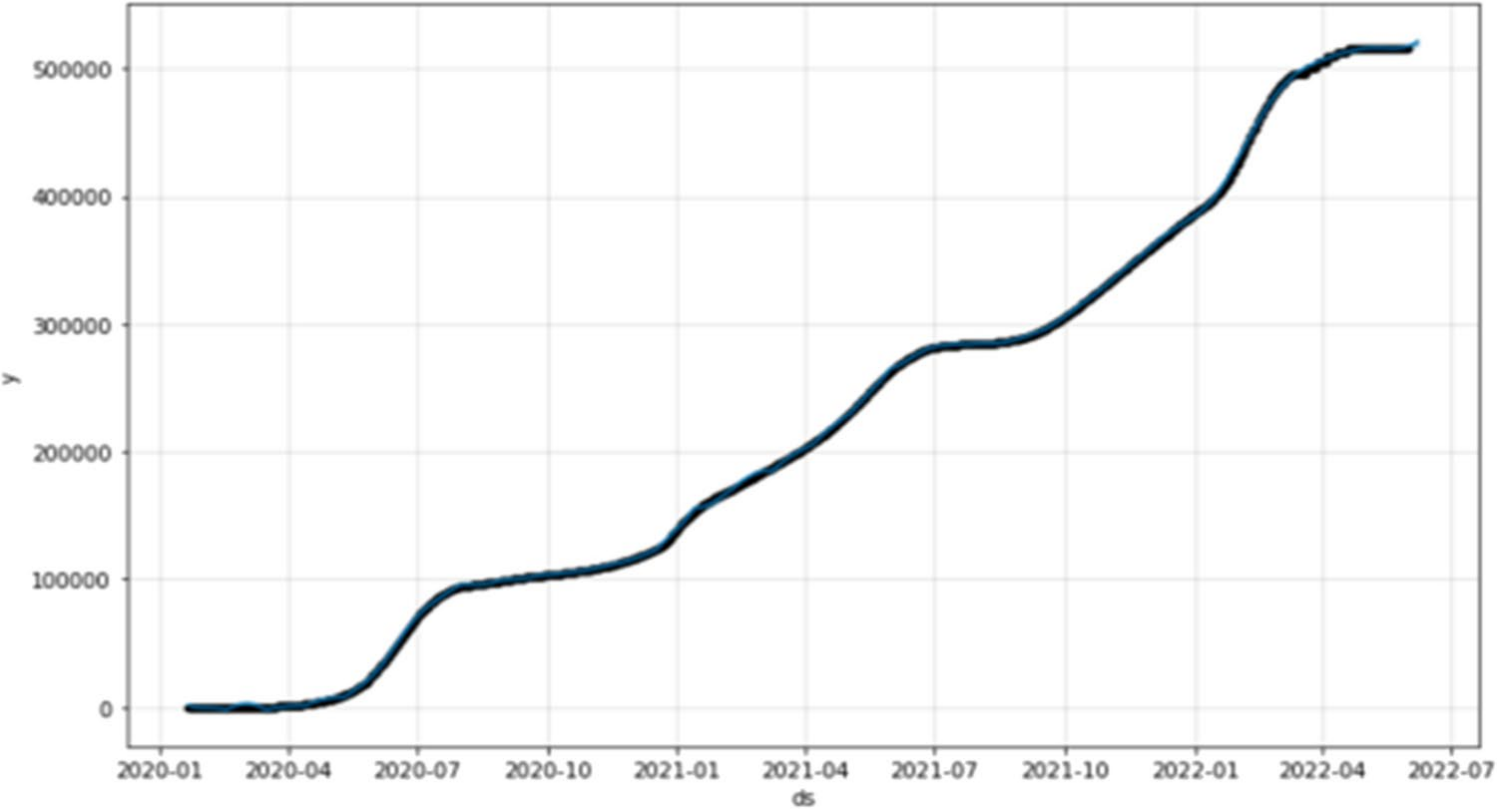
The forecasting curve for confirmed cases

The following Fig. 6 shows the forecasting curve for the next 7 days.

**Fig. 7:**
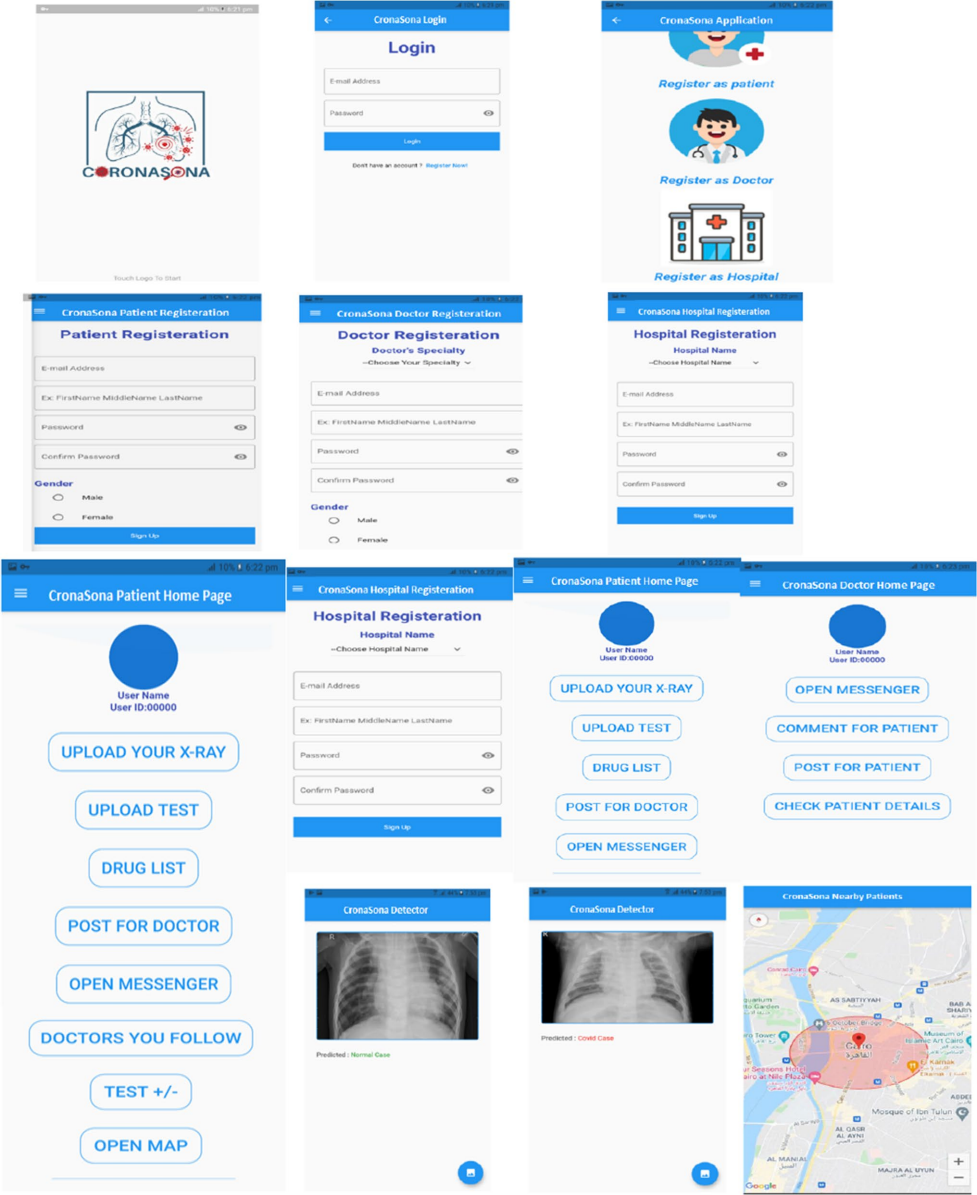
CronaSona interface design

The following Fig. 7 shows the CronaSona interface design.

## Conclusion

In this paper, a healthcare ecosystem framework was introduced to help people face the dangerous COVID-19 epidemic. The proposed framework was designed for COVID-19 diagnosis and management. It consists of two parts: stakeholders and shared components, including four subsystems: the management information subsystem, expert subsystem, COVID-19 detection and forecasting subsystem, and mobile tracker subsystem. The push to develop mobile applications and eHealth solutions in response to the COVID-19 pandemic has resulted in a diverse range of apps and digital healthcare solutions being created. CronaSona was built based on the proposed framework as a step towards helping Egyptians to understand the prevalence and predict situations in Egypt, helping them to communicate with doctors, spread awareness, raise, and improve their psychological state by spreading the answers and trying to help people to each other in emergency situations.

CronaSona intends to contribute to managing the COVID-19 pandemic by providing a tool for diagnosis, forecasting, and tracking. It offers functionalities for stakeholders like administrators, hospitals, doctors, and patients. It helps the country serve people by directing them to the nearest specialized places available for their sites. This project also gives insight into how the country is performing in terms of curbing the spread and increasing citizen awareness. The accuracy findings show promising results compared to other models. It employs Flutter for cross-platform development, SQLite for the database, Google Maps API for the mobile tracker subsystem, TensorFlow for training classification models, Keras for deep neural networks, and Fbprophet for forecasting based on data from a JSON file.

The CronaSona framework, while promising, has certain limitations that need consideration. These limitations highlight areas for refinement and expansion, ensuring CronaSona’s effectiveness, inclusivity, and adaptability to a wider range of healthcare scenarios beyond the current pandemic. The following points summarize CronaSona Limitations.:

1. Limited dataset variability: The effectiveness of the deep learning models used in CronaSona heavily depends on the quality and diversity of the dataset. If the dataset used for training is not representative of all possible variations in COVID-19 cases, the model may not perform well on certain types of cases.
2. Dependency on chest X-rays: CronaSona primarily relies on chest X-rays for COVID-19 detection. While chest X-rays can provide valuable information, they may not be as comprehensive as other diagnostic methods like CT scans and MRI. The framework might benefit from incorporating additional imaging modalities for a more thorough analysis.
3. Accuracy and generalization: Although the accuracy of the CronaSona model is highlighted, its generalization to different populations and geographical locations should be carefully assessed. Variations in demographics, healthcare practices, and COVID-19 strains may affect the model’s performance.
4. Mobile tracker reliability: The effectiveness of the mobile tracker subsystem is contingent on the willingness of individuals to share their location data. Privacy concerns and data security issues might hinder the widespread adoption of this feature.
5. Limited stakeholder roles: The framework’s stakeholders are currently limited to administrators, hospitals, doctors, and patients. Including additional roles such as nurses, caregivers, and public health officials could enhance the system’s versatility and usefulness.
6. Forecasting uncertainties: The accuracy of COVID-19 forecasting is subject to various uncertainties, including changes in virus strains, vaccination rates, and public health interventions. Communicating the inherent uncertainties in forecasting is crucial to managing expectations.
7. Real-world implementation challenges: Deploying CronaSona in the real world involves challenges such as user adoption, integration with existing healthcare systems, and adherence to regulatory and ethical standards. These challenges can affect the successful implementation of the framework.
8. Language and cultural considerations: As CronaSona aims to be a global application, language and cultural differences should be addressed to ensure effective communication and user engagement across diverse populations.
9. Continuous improvement and updates: The dynamic nature of the COVID-19 pandemic requires continuous updates to the framework to adapt to emerging trends, medical knowledge, and technological advancements. Regular maintenance and improvement are essential for the sustained relevance of CronaSona.
10. Global coverage and accessibility: Ensuring CronaSona’s accessibility in regions with limited internet connectivity or technological infrastructure is crucial for its global impact. Addressing disparities in technology access is vital for the framework’s effectiveness worldwide.
11. Testing and experimentation: The application needs extensive testing to ensure its functionality, and experiments should be conducted to validate its performance.
12. Generalization: The overall framework should be generalized to detect and address a broader range of diseases beyond COVID-19.

The proposed CronaSona framework exhibits several improvements compared to similar studies, in the field of COVID-19 detection and management considering various aspects:

1. Integrated healthcare ecosystem framework: CronaSona offers an integrated healthcare ecosystem framework that combines various components, including stakeholders, shared resources, and multiple subsystems. This holistic approach ensures a well-rounded and interconnected system for managing COVID-19 cases and enhances the overall functionality of the application.
2. Mobile tracker subsystem: The inclusion of a mobile tracker subsystem, utilizing Google Maps, adds a unique feature to CronaSona. This subsystem enables the tracking of individuals’ locations to identify potential COVID-19 carriers, providing valuable information to healthy individuals to make informed decisions about their movements, aiding in proactive measures and minimizing virus spread.
3. Expert subsystem and knowledge acquisition: The incorporation of an expert subsystem involves gathering knowledge from both experts and the detection and forecasting subsystem. The expert subsystem involves knowledgeable individuals, such as doctors and knowledge engineers, contributing to the development and validation of the knowledge base. This dual-source knowledge acquisition contributes to ensure a reliable source of information, advice for users within the system, and a richer and more diverse knowledge base, enhancing the accuracy and reliability of the system.
4. Forecasting capability: CronaSona incorporates a COVID-19 detection and forecasting subsystem with two phases—detection of COVID-19 from chest X-ray images and forecasting of future cases. The forecasting module employs machine learning models and is based on data from the World Health Organization, providing valuable insights.
5. User interface enhancements: The user interface of CronaSona includes various functionalities tailored to different stakeholders (admin, hospital, doctor, and patient). This customization improves user experience and ensures that each user group can easily access relevant information and perform necessary actions within the app.
6. Cross-platform development and technology stack: CronaSona is developed using Flutter, a cross-platform framework, making it accessible on various devices. This choice enhances the scalability and reach of the application, potentially benefiting a broader user base. The use of SQLite for database management, Google Maps for location tracking, Tensorflow and Keras for machine learning, and Fbprophet for forecasting represents a robust and diverse technology stack. This choice of technologies enhances the scalability and efficiency of the CronaSona application.
7. Real-time data management: The management information subsystem efficiently manages real-time data from various sources, providing users with accurate and up-to-date information. This real-time data management contributes to better decision-making and responsiveness to changing situations.
8. Comprehensive functionalities: CronaSona offers a range of functionalities for different stakeholders, including administrators, hospitals, doctors, and patients. From data manipulation to communication features and tracking nearby patients, the application serves as a versatile tool for various users within the healthcare ecosystem.
9. Comprehensive testing and experimentation: The proposed future work includes a commitment to conduct comprehensive tests and experiments. This commitment to ongoing improvement and refinement is crucial for ensuring the effectiveness and accuracy of the CronaSona framework.
10. Machine learning models and performance: The implementation of machine learning models, including deep learning algorithms, is a key strength. The paper highlights the performance metrics of the proposed model, demonstrating its accuracy, F1-score, recall, and precision. The model, known as CronaSona, demonstrates superior accuracy (97%) compared to other state-of-the-art deep learning algorithms, as indicated in the presented results. This transparency in reporting results contributes to the credibility of the framework.
11. Future-oriented approach: The proposed method outlines a roadmap for future work, emphasizing continuous improvement and addressing potential limitations. The focus on testing, stakeholder expansion, subsystem enhancements, and generalization for other diseases demonstrates a forward-looking and adaptive approach.
12. Application in a specific context: CronaSona is tailored for the Egyptian context, addressing the specific needs and challenges faced by the population. This localized approach increases the relevance and effectiveness of the application in the given region.

The proposed CronaSona framework exhibits several limitations, which are:

## Future work

To create comprehensive apps with numerous useful features consolidated in one location and supported by trustworthy data and frequent updates, more advancements are required. Strengthening the partnerships between academic and research institutions, healthcare providers, and government entities is essential to developing apps that enhance the quality of public information and bridge the gaps in healthcare exposed by the ongoing pandemic. This scoping assessment highlights the current domains in which COVID-19 apps are being developed and identifies areas for improvement and innovation. Collaborative efforts among key stakeholders will not only provide vital platforms for addressing the present crisis but also establish the groundwork for addressing future health challenges by building robust infrastructure. For that, several improvements might be made to the existing framework and the implemented application. CronaSona has to be subjected to a range of tests and experiments. Stakeholders need to be increased to cover different roles, such as nursing and/or adding a caregiver for patients. The management information subsystem needs to be improved by allowing users to register beds in hospitals. The detection and forecasting subsystem needs to be enhanced by training different DL models to improve performance based on different types of radiomics, such as CT scans and/or MRI. The overall framework needs to be generalized to detect other diseases. In addition, CronaSona needs to be a global application with different languages to cover different countries. The following points stats several strategies and considerations that can be implemented:

1. Enhanced stakeholder roles: CronaSona wishes to consider expanding the roles of stakeholders to include additional healthcare professionals such as nurses and caregivers. It also needs to integrate features that allow caregivers to support patients and communicate with healthcare providers.
2. Improved data quality and quantity: Ensure a diverse and comprehensive dataset, including high-quality chest X-ray images and collaborate with healthcare institutions and organizations to access a larger and more representative dataset.
3. Multimodal approaches: Explore the integration of multiple modalities such as chest X-rays, CT scans, and clinical data for a more comprehensive understanding of COVID-19 cases and investigate the use of advanced imaging techniques to enhance diagnostic capabilities.
4. Improvements in management information subsystem: CronaSona needs to enable users to register available beds in hospitals through the management information subsystem. It also needs to implement features that provide real-time updates on hospital capacity and resource availability.
5. Enhanced detection and forecasting subsystem: CronaSona deep learning models need to be trained on diverse datasets, including different types of radionics such as CT scans and MRI, to improve performance across various medical imaging modalities. Additionally, it needs to implement continuous learning mechanisms to adapt the models based on emerging patterns in the data.
6. Explainability and interpretability: Utilize interpretable machine learning models and techniques to enhance user trust and implement models that provide clear explanations for their predictions, ensuring transparency in decision-making processes.
7. Globalization and multilingual support: The future vision for CronaSona includes global application by incorporating multilingual support, aiming to cater to diverse populations and regions and considers collaborating with international healthcare organizations to ensure the framework is adaptable to diverse healthcare systems.
8. Usability and user interface enhancements: Conduct user testing to gather feedback on the user interface and overall usability then implement improvements based on user feedback to enhance the overall user experience.
9. Collaboration with healthcare institutions: Strengthen partnerships with academic and research institutions, healthcare providers, and government entities to ensure the framework is aligned with the latest healthcare standards and guidelines. In addition, CronaSona needs to establish a mechanism for regular updates and collaboration to address evolving healthcare challenges.
10. Comprehensive testing and experimentation: Conduct experiments to validate the effectiveness of the forecasting models and make adjustments based on the results. Additionally, focus on security audits and performance testing, to ensure robustness and data security.
11. Generalization for other diseases: Explore the possibility of generalizing the framework to detect and manage other diseases, expanding its utility beyond COVID-19.
12. Community engagement and awareness: Implement features that facilitate community engagement and awareness, encouraging users to actively participate in the collective effort to combat the pandemic, and incorporate educational resources to raise awareness about preventive measures and public health guidelines.
13. Continuous improvement and updates: Establish a system for regular updates and continuous improvement based on emerging healthcare trends and user feedback. Stay informed about advancements in technology and healthcare practices to incorporate the latest innovations into the framework.

## Data Availability

The datasets used during the current study are available in the [Kaggle] repository https://www.kaggle.com/datasets/paultimothymooney/chest-xray-pneumonia.

## Footnotes

1 https://www.worldometers.info/coronavirus/, accessed December 6, 2023.

2 https://www.kaggle.com/datasets/paultimothymooney/chest-xray-pneumonia
